# Phenolic profile and antioxidant potential of wild watercress (*Nasturtium officinale* L.)

**DOI:** 10.1186/s40064-015-1514-5

**Published:** 2015-11-24

**Authors:** Alam Zeb

**Affiliations:** Department of Biotechnology, University of Malakand, Chakdara, Pakistan

**Keywords:** Watercress, Antioxidant activity, Pigments, Total phenolic contents, Phenolic compounds

## Abstract

Phenolic profile, antioxidant potential and pigment contents of wild watercress (*Nasturtium officinale* L.) were studied to assess the potential for future studies and its applications in neutraceuticals and bioactive functional ingredients. Different extracts of watercress (roots, stem and leaves) were analysed for pigment composition, total phenolic contents, and radical scavenging activity. The phenolic profile of the leaves and roots was studied using reversed phase HPLC–DAD. Results showed that total phenolic compounds in all samples were higher in the methanolic extracts than its corresponding aqueous extracts. The RSA of methanolic extracts was higher than aqueous extracts. Fourteen phenolic compounds were identified in the leaves, where coumaric acid and its derivatives, caftaric acid and quercetin derivatives were present in higher amounts. In roots, a total of 20 compounds was tentatively identified, with coumaric acid and its derivatives, sinapic acid, caftaric acid and quercetin derivatives were the major phenolic compounds. In conclusion, watercress has significant antioxidant activity and contains important phenolic compounds, which could be of potential biological interest.

## Background

Watercress (*Nasturtium officinale* L.) is an aquatic perennial plant that belongs to Brassicaceae family. It is a leafy vegetable found in and around water. The taste of the leaves is like strong pepper, which leads to its commercial use in salads. The native lands of this plant are western Asia, India, Europe and Africa (Cruz et al. 2008). It is generally found in bunches in cold, mildly flowing, and low stream. It naturally grows in brooks, ditches and pond margins. The plant is cultivated in lakes, ponds and in slow-moving water in rivers, canals and streams. It is a significant part of water ecosystems. It provides good habitation for many aquatic organisms and protection for young fish and amphibians (Rose et al. 2000). The presence of different phytochemicals and nutritional benefits of watercress make it a healthy diet that maintains the immunity and health of the body. As a salad vegetable, Watercress provides sufficient nutrients. Pigments such as carotenoids and chlorophyll have significant potential of antioxidant activity. The therapeutic capability of carotenoids includes cancer inhibition (Seifried et al. 2003), effective in cardiovascular problems (Granado et al. 2003), enhances the function of the immune systems and is effective against aging-eye diseases and against free radical reactions (Zeb and Murkovic 2011). Watercress is considered as an important source of carotenoids, which are consumed in diets (Hart and Scott 1995).

Watercress supplementation in diet has shown to ameliorate the DNA damage and increased the blood antioxidant potential in human subjects (Gill et al. 2007). The increase in the blood antioxidant potential may be due to the presence of glucosinolate contents in the blood (Dyba et al. 2010). Watercress consumption has strong potential to act as a source of anti-cancer drug (Hecht et al. 1995) and its extract provides protection against the genotoxins at the different cancer stages (Boyd et al. 2006). Watercress was found to contain several phenolic compounds that contributed to the anti-cancer potential (Ozen 2009). Martínez-Sánchez et al. (2008) showed that the flavonoids in watercress leaves were rich in a characteristic glycosylation pattern with rhamnose at the 7 position. Similarly, Aires et al. (2013) studied the phenolic compounds and antioxidant activity of the watercress baby-leaves. Santos et al. (2014) revealed the phenolic profile of cultivated variety of watercress bay-leaves along with other leafy vegetables. The authors identified 19 compounds from the leaves. These studies, however, lack information about the phenolic composition and antioxidant potential of wild watercress and its roots. The present study was therefore aimed to determine the pigment composition, antioxidant activities and phenolic profile of the leaves and roots of wild grown watercress.

## Results and discussion

### Pigments composition

**Fig. 1:**
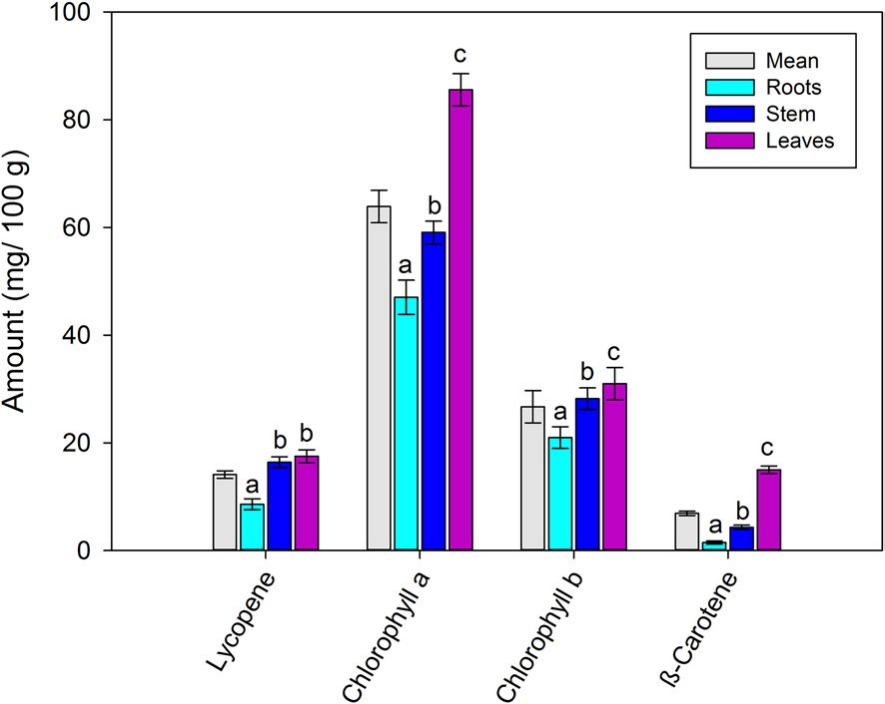
Pigment composition of watercress roots, stem and leaves. *Different letters* in same pigment represent significant at p < 0.05

The pigments profile was determined in the different parts of the watercress is shown in Fig. 1. Lycopene in root, stem and leaves of watercress methanolic extract were 8.6, 16.4 and 17.5 mg/100 g, respectively. Chlorophyll *a* in root, stem and leaves of watercress methanolic extract was 47.03, 59.1 and 85.6 mg/100 g, respectively. There was a significant increase (p < 0.05) in the chlorophyll *a*, which was in order of root < stem < leaves. Chlorophyll *b* present in the watercress methanolic extract was 21.0, 28.2 and 31.0 mg/100 g in root, stem and leaves, respectively. The increase was significant (p < 0.05) in chlorophyll *b* in order root < stem < leaves. The β-carotene was present in the root, stem and leaves of watercress methanolic extract was 1.5, 4.3 and 15.0 mg/100 g, respectively. The significant (p < 0.05) increase in β-carotene was in the order of root < stem < leaves. Sunlight on the leaves stimulate the photosynthetic reactions and formation of phenolic compounds (Vidovic et al. 2015). There are several internal and external factors, which are involved in the metabolism of these photosynthetic pigments (Esteban et al. 2015), especially the amount of these pigments. These results indicate that the total amount of chlorophyll *a* and *b* was higher in all tissue samples than the other pigments.

### Total phenolic contents

**Fig. 2:**
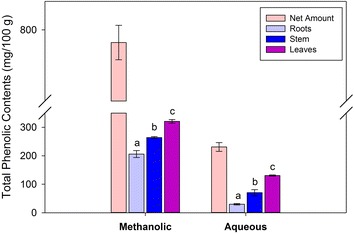
Total phenolic contents (TPC) of methanolic and aqueous extracts of watercress roots, stem and leaves. *Different letters* in same extract represent significant at p < 0.05

Phenolic compounds are soluble both in water and organic solvent such as methanol. Therefore, two extractions were carried out using water and methanol. The standard calibration curves were prepared separately for aqueous and methanolic extracts. The standard gallic acid calibration curve for aqueous extracts have a concentration range of 0.5–5 mg/mL with equation of y = 0.0391 x + 0.1324. Similarly, the second calibration was prepared for methanolic extracts using 1–20 mg/mL concentration with calibration equation of y = 0.0324 x + 0.2503. Total phenolic content in root, stem and leaves of watercress aqueous extract were 29.8, 70.4 and 130.8 mg of GAE/100 g, respectively (Fig. 2). The total phenolic content in the root, stem and leaves of aqueous extract of watercress ranged from 161.2 to 231.0 mg of GAE/100 g of dry weight. The TPC in aqueous extract has a significant (p < 0.05) increase in the order root < stem < leaves. In methanolic extracts of the root, stem and leaves, the TPC were 205.99, 264.0 and 321.1 mg of GAE/100 g, respectively. These results indicate that TPC were higher in the methanolic extract than aqueous extract. These results were in agreement with Abdul et al. (2014), who reported that TPC in the methanolic extract was higher (121.4 mg GAE/g) than its corresponding aqueous extracts (99.2 mg GAE/g). The TPC of the leaves lower than the values (1400 mg/100 g) reported recently (Aires et al. 2013). The difference may be due to the method of extraction, variety of plant and environmental factors.

### Radical scavenging activity (RSA)

**Fig. 3:**
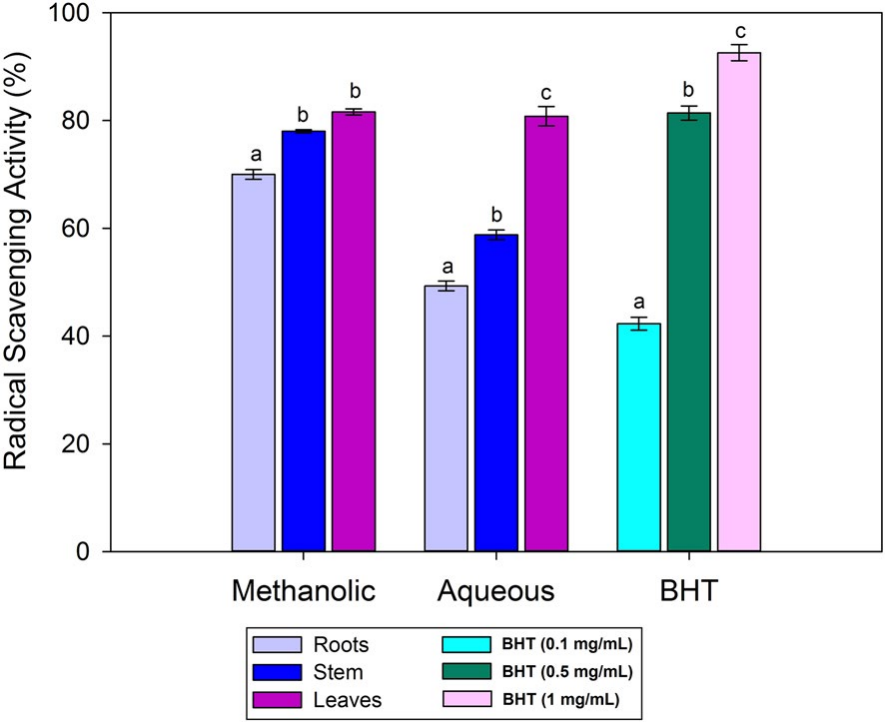
Radical scavenging activity of methanolic and aqueous extract of watercress roots, stem and leaves and BHT. *Different letter* in same extraction represent significant at p < 0.05

The radical scavenging activity (RSA) of root, stem and leaves of watercress methanolic extract were 70.0, 78.0 and 81.6 %, respectively. There was a significant (p < 0.05) increase in % RSA in the methanolic extract with the order of root = stem < leaves. A relatively low % RSA values were obtained for aqueous extracts, which were 49.3, 58.8 and 80.8 %, respectively. The results of the RSA values of the both extracts were in close agreement with RSA values of BHT (0.5 mg/mL) as shown in Fig. 3. Abdul et al. (2014), showed that the DPPH radical scavenging activity of the methanolic leaves extract was lower than its corresponding water extract, while the results of this study showed no significant difference among the two types of extracts. This may be due to the difference in the extraction procedures, purity of the solvents, variety of watercress and seasonal temperatures. There was a significant (p < 0.05) increase in the % RSA activity, which was in the order of root < stem < leaves (Fig. 3). The amount of RSA was, however, relatively similar in the aqueous and methanolic extracts of the leaves. The result of aqueous extract RSA value is comparable with results of Ozen (2009), who showed that the aqueous extract of watercress leaves had similar antioxidant potential with that of tocopherol.

### Phenolic compounds in watercress leaves

**Fig. 4:**
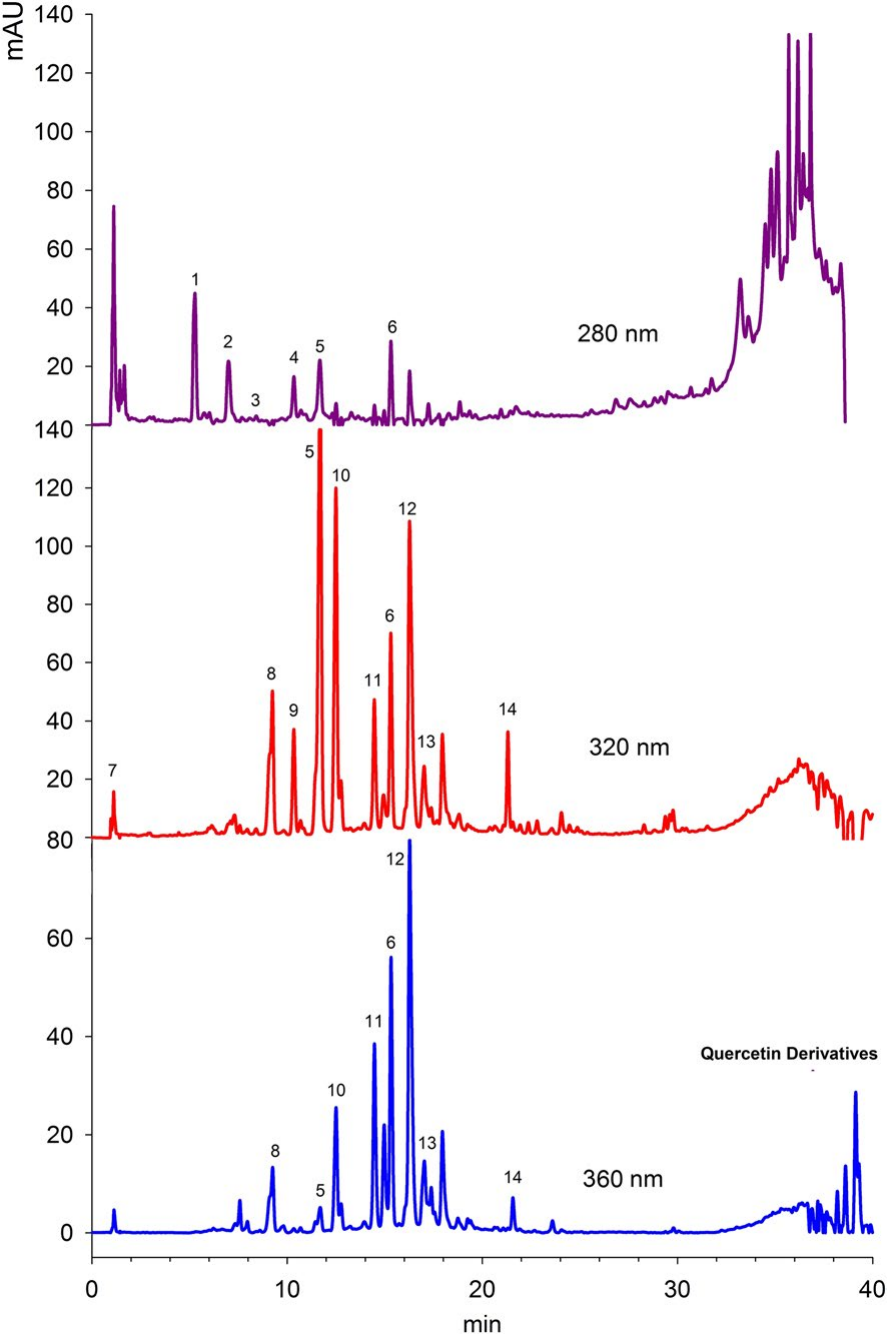
Typical chromatograms of watercress leaves at 280, 320 and 360 nm. *Each peak* represent individual phenolic compound, with the details as explained in Table 1

Phenolic compounds in watercress leaves were determined using three wavelengths, i.e. 280, 320 and 360 nm as shown in Fig. 4. Each peak no in the chromatograms represents individual compounds, which are explained in Table 1. Only those compounds were taken into consideration, whose purity was higher than 99.0 %. At 280 nm, six phenolic compounds were tentatively identified. Peak 1 and 2 was identified as gallic acid derivatives. Aires et al. (2013) had also identified gallic acid in watercress leaves. Peak 3 was found to be Ferulic acid derivatives, while peak 4 and 5 was Pro-anthocyanidin B1 and *p*-coumaric acid derivatives. The exact structure of these compounds was not identified because of the lack of mass spectrometric detection as well as reference data in the literature. Peak 6 was identified as Apigenin with characteristic absorption maxima at 336 and 276. At a wavelength of 320, a total of 10 compounds has been identified. Compound 7 was *p*-hydroxybenzoic acid, compound 8 was sinapic acid, and compound 9 was *p*-coumaric acid. Caftaric acid was spotted at peak 10 with retention time of 12.5 min, while compound 11 and 12 was identified as Quercetin-3-(caffeoyldiglucoside)-7-glucoside and caffeoylmalic acid. These compounds eluted at 16.2 and 17.0 min and was also identified by Santos et al. (2014). However, these authors identified all compounds using 280 nm, while in this study, some new compounds such as ferulic acid, Pro-anthocyanidin B1, apigenin and caftaric acid were reported in watercress leaves for the first time. Peak 14 was identified as coumaric acid derivatives, however the exact nature of the derivative part has not been identified. At 360 nm a total of eight peaks was identified.

**Table 1:** Identification of phenolic compounds in Watercress leaves

Peak	Rt (min)	Identity	HPLC–DAD λ_max_ (nm)	References
1	5.2	Gallic acid derivative	273, 279, 288	Aaby et al. (2007)
2	6.9	Gallic acid derivative	271, 278, 287	Aaby et al. (2007)
3	8.4	Ferrullic acid derivative	328, 300	Aaby et al. (2007)
4	10.3	Proanthocynidin B1	309, 300	Aaby et al. (2007)
5	11.6	*p*-Coumaric acid derivative	313	Santos et al. (2014)
6	15.3	Apigenin	336, 276	Santos et al. (2014)
7	1.1	*p*-Hydroxybenzoic acid	259	Santos et al. (2014)
8	9.2	Sinapic acid	329, 304	Aaby et al. (2007)
9	10.3	*p*-Coumaric acid	309, 300	Santos et al. (2014)
10	12.5	Caftaric acid	328, 298	Aaby et al. (2007)
11	14.4	Quercetin-3-(cafferoyldiglucoside)-7-glucoside	336, 300, 270, 255	Santos et al. (2014)
12	16.2	Kaempferol-3-(caffeoyldiglucoside)-7-rhamnoside	332, 398sh, 268, 255	Aaby et al. (2007)
13	17.0	Caffeoylmalic acid	327, 300, 268	Santos et al. (2014)
14	21.3	Coumaric acid derivative	312, 268	Santos et al. (2014)

Peak no corresponds to the phenolic compounds eluted in the chromatogram (Fig. 4)

### Phenolic compounds in Watercress roots

**Fig. 5:**
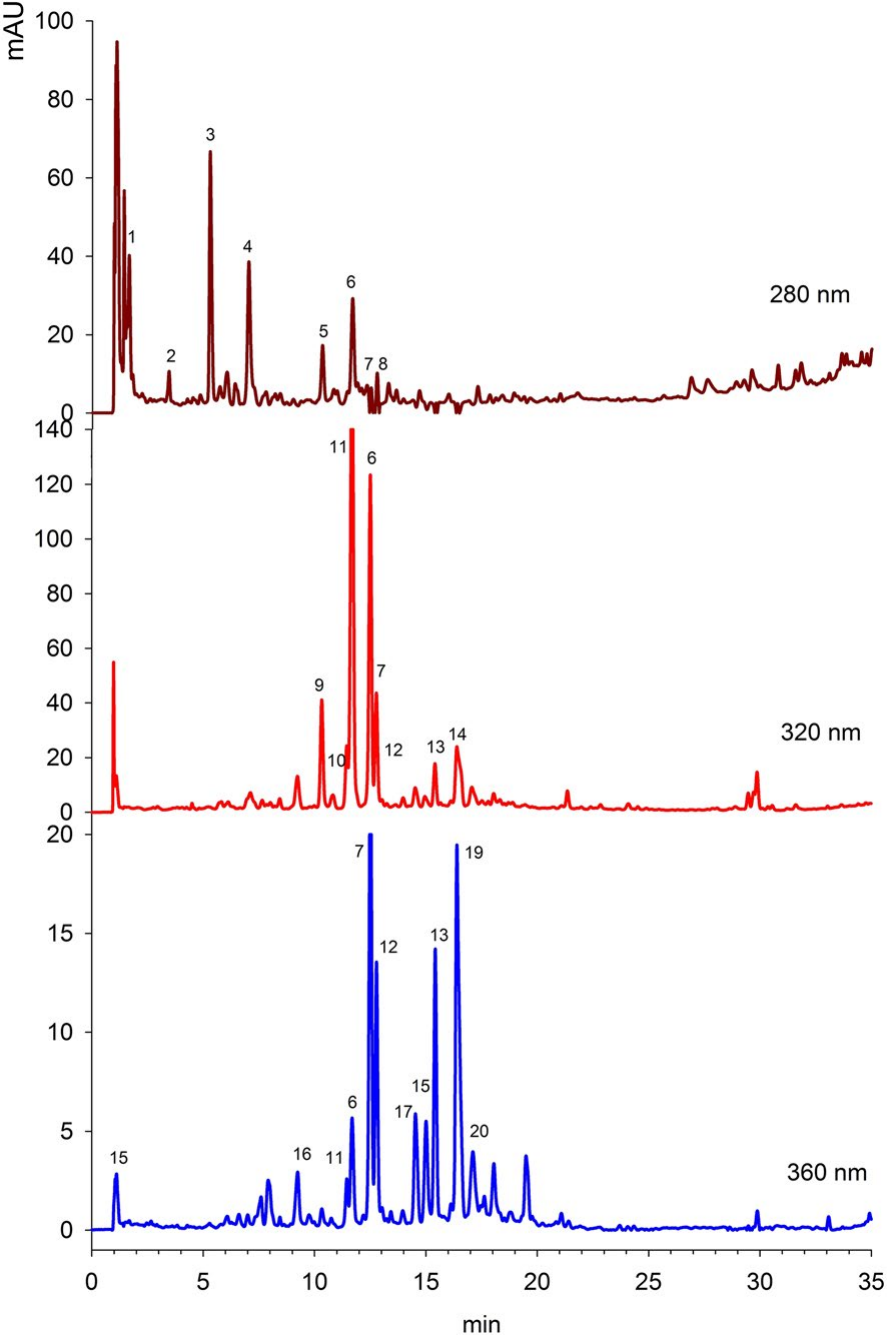
Typical chromatograms of watercress roots at 280, 320 and 360 nm. *Each peak* represent individual phenolic compound, with the details as explained in Table 2

Figure 5 showed the separation of about twenty phenolic compounds in the watercress roots. The tentative identification of these peaks has been shown in the Table 2. Gallic acid was found to elute at 1.6 min and was marked as peak 1. Peaks 2 and 3 were found to be the derivatives of gallic acid and hydroxybenzoic acid, respectively. The identity of other compounds was carried out with reference to the literatures (Aaby et al. 2007; Santos et al. 2014). Peak 5 was identified as *p*-coumaric acid, while peak 6 was its derivative. Caftaric acid and sinapic acid eluted at the 12.4 and 12.7 min, respectively, and were marked as peak 7 and 8. Sinapic acid or its derivatives are characteristic compounds present in large amount in the plants of Brassicaceae family. Sinapic acid have shown to possess antioxidant, antimicrobial, anti-inflammatory, anticancer, and anti-anxiety activity (Niciforovic and Abramovic 2014). Thus, the wild watercress may also possess such activity. In 320 nm, a total of eight compounds was identified together with peak 6 and 7. Peak 9 was found to be Pro-anthocyanidin trimer, while peaks 10 and 11 were caffeic acid hexoside and caffeic acid derivative. Sinapic acid glucoside was identified at 12.7 min with absorption maxima of 328 nm. The highest percent peak areas were obtained for peak 6 and 11. Peaks 13 and 14 were kaempferol-3-(caffeoyldiglucoside)-7-rhamnoside and quercetin-3, 7-diglucoside, respectively. At 360 nm, a total of 11 compounds was identified, including compounds 6 and 7. Compound 15 was identified as hydroxybenzoic acid, and compound 16 as vanillic acid with absorption maxima of 263 and 290 nm. Vanillic acid has been found to protect liver from the chemical induced injury in rats (Itoh et al. 2009). The presence of vanillic acid in watercress may thus be of the potential biological applications. Spinacetin glucuronide was eluted at 14.5 min and marked as peak 17. Peak 18 was dihydro kaempferol hexoside, and compound 20 was obtained in pure peak as quercetin-3-*O*-rutinoside-7-*O*-glucoside. Santos et al. (2014) had also identified this compound in watercress baby-leaves. Compound 20 was tentatively identified as quercetin-3-*O*-triglucoside. Quercetin-3-*O*-triglucoside was also identified in other Brassica family plants (Schmidt et al. 2010). This suggests that quercetin-3-*O*-triglucoside can be used as a marker for the analysis of phenolic compounds in Brassica plants. The highest amount was of compound 7, 12, 13 and 19. Several quercetin derivatives were eluted at later time in a chromatographic run. However, the complete structural identity was not possible with diode array detection only. Quercetin or its derivatives have been found to protect cells from hydrogen peroxide induced cytotoxicity, and results into a decrease in the production of reactive oxygen species (Shokoohinia et al. 2015). Thus, wild watercress is the source of large amount of quercetin or its derivatives with significant antioxidant and biological properties.

**Table 2:** Identification of phenolic compounds in Watercress roots

Peak	Rt (min)	Identity	HPLC–DAD λ_max_ (nm)	References
1	1.6	Gallic acid	270	Santos et al. (2014)
2	3.4	Gallic acid derivative	269, 278	Santos et al. (2014)
3	5.2	Hydroxybenzoic acid derivative	279, 273	Santos et al. (2014)
4	7.0	Gallic acid derivative	271, 277	Santos et al. (2014)
5	10.3	*p*-Coumaric acid	309, 298	Santos et al. (2014)
6	11.6	*p*-Coumaric acid derivative	313	Santos et al. (2014)
7	12.4	Caftaric acid	328, 298	Aaby et al. (2007)
8	12.7	Sinapic acid	329, 304	Aaby et al. (2007)
9	10.3	Pro-anthocynidin trimer	309, 298sh	Aaby et al. (2007)
10	10.8	Caffeic acid hexoside	293, 283	Aaby et al. (2007)
11	11.4	Caffeic acid derivative	321, 296	Santos et al. (2014)
12	12.7	Sinapic acid glucoside	328	Aaby et al. (2007)
13	15.4	Kaempferol-3-(caffeoyldiglucoside)-7-rhamnoside	332, 398sh, 268, 255	Aaby et al. (2007)
14	21.3	Quercetin-3,7-diglucoside	266, 280, 330	Santos et al. (2014)
15	1.1	Hydroxybenzoic acid		Santos et al. (2014)
16	10.1	Vanillic acid	263, 290	Aaby et al. (2007)
17	14.5	Spincetine glucuronide	270, 340	Aaby et al. (2007)
18	15.0	Dihydro kaempferol hexoside	280, 290	Aaby et al. (2007)
19	16.3	Quercetin-3-*O*-rutinoside 7-*O*-glucoside	254, 342	Santos et al. (2014)
20	17.1	Quercetin-3-*O*-triglucoside	268, 340	Santos et al. (2014)

Peak no corresponds to the phenolic compounds eluted in the chromatogram (Fig. 5)

## Conclusions

The antioxidant potential, pigments and phenolic composition of wild watercress leaves and roots was studied for the first time, in order to assess the potential for future studies and applications in neutraceuticals and bioactive functional ingredients. Among the pigments, chlorophyll a was found in highest amount. Total phenolic compounds were higher in the methanolic extract than aqueous extract. The radical scavenging activity of methanolic extracts was higher than its corresponding aqueous extracts. A total of 14 phenolic compounds was identified in the leaves, where coumaric acid and its derivatives, caftaric acid and quercetin derivatives were present in higher amounts. In roots, a total of 20 compounds was tentatively identified, with coumaric acid and its derivatives, caftaric acid and quercetin derivatives were the major phenolic compounds. This study showed the presence of some new compounds that were not reported previously. The complete phenolic profile will only be possible with uses of mass spectrometry. Thus, it is concluded that watercress leaves and roots are a good source of bioactive compounds.

## Methods

### Chemicals and reagents

Gallic acid was purchased from BDH (BDH, England), methanol was from Sigma-Aldrich (Steinheim, Germany). All other chemicals and reagents were of analytical standards.

### Samples collection and preparation

The plant samples of wild watercress were collected from the grown at the river side of Lower Dir, Khyber Pakhtunkhwa, Pakistan. The samples were collected in May 2014. Three parts of the samples leaves, stems and roots were separated. The samples were a shade dried and grinded to fine powder. The powder samples were then stored in refrigerator until analysis. Two extracts i.e. methanolic and aqueous were prepared.

### Determination of pigments

One gram of a dried powder sample was taken and mixed with 20 mL of acetone and hexane (4:6, v/v). The mixture was vigorously shaken for 10 h and filtered with Whatman No. 1 filter paper. Pigments were measured using the method of Nagata and Yamashita (1992). The absorbance of the extract was measured at 453, 505, 645 and 663 nm using UV–visible spectrophotometer, model PharmaSpec UV-1700 (Shimadzu, Japan). Lycopene content was calculated at according to the formula: Lycopene (mg/100 mL) = −0.0458 × A663 + 0.204 × A645 − 0.304 × A505 + 0.452 × A453; Chlorophyll *a* (mg/100 mL) = 0.999 × A_663_ − 0.0989 × A_645_; Chlorophyll *b* (mg/100 mL) = −0.328 × A_663_ + 1.77 × A_645_; beta-carotene (mg/100 mL) = 0.216 × A_663_ − 1.220 × A_645_ − 0.304 × A_505_ + 0.452 × A_453_, and the results are further expressed in mg/100 mL of dry weight.

### Total phenolic contents

Total phenolic contents (TPC) were determined using Folin-Ciocalteu (FC) reagent. The samples were mixed with 2 mL FC reagents and 2 mL Na_2_CO_3_ (7.5 %). The mixture was taken in the test cuvettes and incubated for one hour. UV–visible spectrophotometer (Shimadzu, Japan) was used for the measurement of the absorbance at 765 nm (Zeb and Ullah 2015). The standard calibration curve was prepared separately for both aqueous extract and methanol extract. The TPC from methanol extract was determined from the calibration curve (y = 0.0324x + 0.2503, R^2^ = 0.9699), and the TPC from aqueous extract was determined from the second calibration curve (y = 0.0391x + 0.1342, R^2^ = 0.9357). The result was expressed in mg of Gallic acid in 100 g of sample powder.

### Radical scavenging activity (RSA)

Radical scavenging activity (RSA) was determined using DPPH free radicals of method reported by Zeb and Mehmood (2012) with little modification. The DPPH solution of 0.1 mM, BHT standards (0.1, 0.5 and 1.0 mg/mL) and sample extracts were prepared in methanol. The absorbance was measured at 515 nm with the help of Shimadzu PharmaSpec 1770 (Shimadzu, Japan) spectrophotometer. The % RSA was calculated from the absorbance of the control and extract samples.

### HPLC–DAD analyses of phenolic compounds

Phenolic compounds were extracted from the powder samples using the modified method reported previously (Pirisi et al. 2000). Briefly 1 g of the powder sample was dissolved in 10 mL methanol–water (60:40) mixture and vigorously shaken for 10 h. The mixture was filtered and centrifuge for 10 min at 4000 rpm. The samples were then filtered and injected into the HPLC system.

The phenolic compounds were separated using Agilent 1260 Infinity HPLC system consists of quaternary pump, degasser, auto-sampler and diode array detector (DAD). The separation was achieved with the help of Agilent Rapid Resolution Zorbax Eclipse plus C18 (4.6 × 100 mm, 3.5 µm) column, which was maintained at 25 °C. The gradient system consists of solvent A (methanol:acetic acid:deionized water, 10:2:88) and solvent B (methanol:acetic acid:deionized water, 90:2:8) (Zeb 2015). The gradient program was started with 100 % A at 0 min, 85 % A at 5 min, 50 % A at 20 min, 30 % A at 25 min, and 100 % B from 30 to 40 min. The flow rate was 1 mL/min. The chromatograms were obtained using 280, 320 and 360 nm for analysis of phenolic compounds. The spectra were recorded from 190 to 450 nm. The identification was carried out using available standard, its retention times, or the UV absorption spectra from the reported literature.

### Data analysis

All samples were measured in triplicate. Data were analysed for variation by one way analysis of variance (ANOVA) at α = 0.05 using Sigma Plot for windows version 12.0 (Systat Software Inc, 2013).

## Abbreviations

TPC: total phenolic contents
HPLC-DAD: high performance liquid chromatography with diode array detector
RSA: radical scavenging activity
DNA: deoxyribonucleic acid
UV: ultraviolet
GAE: gallic acid equivalents
